# Multiple-Omics Techniques Reveal the Role of Glycerophospholipid Metabolic Pathway in the Response of *Saccharomyces cerevisiae* Against Hypoxic Stress

**DOI:** 10.3389/fmicb.2019.01398

**Published:** 2019-06-27

**Authors:** Zhengchao Xia, Xuelin Zhou, Jingyi Li, Lei Li, Yi Ma, Yi Wu, Zhong Huang, Xiaorong Li, Pingxiang Xu, Ming Xue

**Affiliations:** ^1^Department of Pharmacology, Beijing Laboratory for Biomedical Detection Technology and Instrument, School of Basic Medical Sciences, Capital Medical University, Beijing, China; ^2^Beijing Tropical Medicine Research Institute, Beijing Friendship Hospital, Capital Medical University, Beijing, China; ^3^Central Laboratory, Capital Medical University, Beijing, China; ^4^Health Branch College, Lanzhou Modern Vocational College, Lanzhou, China; ^5^Beijing Engineering Research Center for Nerve System Drugs, Beijing, China

**Keywords:** hypoxia, transcriptomics, proteomics, metabolomics, *Saccharomyces cerevisiae*, glycerophospholipid metabolism

## Abstract

Although the biological processes of organism under hypoxic stress had been elucidated, the whole physiological changes of *Saccharomyces cerevisiae* are still unclear. In this work, we investigated the changes of biological process of *S. cerevisiae* under hypoxia by the methods of transcriptomics, proteomics, metabolomics, and bioinformatics. The results showed that the expression of a total of 1017 mRNA in transcriptome, 213 proteins in proteome, and 51 metabolites in metabolome had been significantly changed between the hypoxia and normoxia conditions. Moreover, based on the integration of system-omics data, we found that the carbohydrate, amino acids, fatty acid biosynthesis, lipid metabolic pathway, and oxidative phosphorylation were significantly changed in hypoxic stress. Among these pathways, the glycerophospholipid metabolic pathway was remarkably up-regulated from the mRNA, protein, and metabolites levels under hypoxic stress, and the expression of relevant mRNA was also confirmed by the qPCR. The metabolites of glycerophospholipid pathway such as phosphatidylcholine, phosphatidylethanolamine, phosphoinositide, and phosphatidic acids probably maintained the stability of cell membranes against hypoxic stress to relieve the cell injury, and kept *S. cerevisiae* survive with energy production. These findings in the hypoxic omics and integrated networks provide very useful information for further exploring the molecular mechanism of hypoxic stress.

## Introduction

Oxygen plays an important role in the survival of many eukaryotic cells. Hypoxia of cells, tissues, organs, and organisms causes a series of pathophysiological processes and plays important role in different diseases such as altitude sickness, stroke, asthma, angina pectoris, myocardial infarction, and tumor (Stub et al., 2015; McBrien et al., 2018). The budding yeast, *Saccharomyces cerevisiae*, has widely used as a powerful eukaryotic model to elucidate the protective mechanism of organism in various of stresses, including hypoxia stress (Hess et al., 2014). In the previous studies, the analysis of *S. cerevisiae* in hypoxia stress showed that the oxidoreductase and the metabolites of respiratory chain were changed (Vukotic et al., 2012). In recent years, the study of *S. cerevisiae* in the biological process involved in the change of glycolysis (Jin et al., 2017), lipid metabolism (Degreif et al., 2017), mitophagy (Liu et al., 2014), etc.

The omics technologies were more and more applied on *S. cerevisiae* because of the innovation of new instrument, technique, and bioinformatics analysis methods (Liu et al., 2017; Lapointe et al., 2018). Chromatography (e.g., GC and HPLC) coupled with mass spectrometry also accelerates the analysis process on *S. cerevisiae* (Searle et al., 2018). Transcriptomics is used to investigate the transcription levels of genes in different stresses to elucidate the regulated mechanism. Especially, a novel high-throughput sequencing approach (RNA-seq) provides more precise and comprehensive measurement in transcripts and their isoforms (Cheng et al., 2018; Hu et al., 2019). Proteomics aims to find out the vital target proteins with different methods such as iTRAQ, SILAC, and label free, which explore their functions or post-translational modification (Cui et al., 2015; Bechtner et al., 2019). Metabolomics serves as a key relation between genotype and phenotype and focuses on the analysis of endogenous and exogenous small molecules (Zhou et al., 2015). Metabolomics is probably the most frequently used omics technique to discover the potential biomarkers under the numerous diseases and study the metabolic flux on *S. cerevisiae* (Abecia et al., 2018; Farne et al., 2018).

These omics could obtain respective partial data, but these individual data were difficultly integrated to clarify the life process from gene, protein, and metabolite (Ghaemi et al., 2019). In the same way, although the system-omics of *S. cerevisiae* had existed (Braconi et al., 2016; Lapointe et al., 2018), the adaptation mechanism of *S. cerevisiae* in hypoxia stress was still unclear, especially at metabolic pathway levels of the gene, protein, and metabolites. Our work used multiple-omics techniques to acquire more fruitful data with appropriate bioinformatics tools and elucidate the adaptation mechanism of *S. cerevisiae* under hypoxia.

## Materials and Methods

### Cell Culture

*Saccharomyces cerevisiae* was purchased from China General Microbiological Culture Collection (CGMCC) as a freeze-dried powder in a glass vial. The *S. cerevisiae* strains were grown in YPD medium (including 1% [w/v] peptone, 0.5% [w/v] *S. cerevisiae* extract and 1% [w/v] glucose) at 28°C in a three-gas cell culture incubator. *S. cerevisiae* was incubated for 24 h and stored at −80°C supplemented with same volume of 50% glycerol for seed conservation.

### Hypoxic Conditions

The seed of *S. cerevisiae* was inoculated into YPD medium at a ratio of 1:100 in the culture flask and continuously filled with high purity nitrogen gas to sustain hypoxic circumstance in cell culture incubator. The oxygen concentrations of normoxic group and hypoxic groups were 21% (Con21), 10% (Hpx10), 5% (Hpx5), and 1% (Hpx1), respectively. The absorbance values were determined at 600 nm at the intervals of 2 h by spectrophotometry (Spectramax M2, United States) from three replicated cultures. The corresponding growth curve of *S. cerevisiae* was also plotted. Pelleted cells from the triplicate cultures were frozen in liquid nitrogen and stored at −80°C before use.

### Determination of Relevant Enzymes Activities

*Saccharomyces cerevisiae* was incubated for 24 h at different oxygen concentrations and centrifuged at 1000 *g* for 5 min at 4°C. The pellet was washed by phosphate buffer saline (PBS) and sonicated by ultrasonic cell disruptor at 200 W for 4 min. Then the samples were centrifuged at 13,000 *g* for 20 min at 4°C to obtain the supernatant. These enzymes activities of CS, SOD, and ADH were determined by the different kits (Nanjing Jiancheng Bioengineering Institute, China).

### Transcriptomics Analysis

Cells were cultured as mentioned above and their total RNA was extracted using the mirVana miRNA isolation kit (Ambion) as described by the manufacturer. RNA integrity was evaluated using Agilent 2100 Bioanalyzer. All samples with RNA integrity number (RIN) more than 7 were subjected to the subsequent analysis. The RNA sequencing (RNA-Seq) libraries were constructed using TruSeq Stranded mRNA LTSample Prep Kit (Illumina) following the manufacturer’ s instructions. In addition, these libraries were sequenced on the Illumina sequencing platform (HiSeqTM 2500) and 125 bp paired-end reads were generated.

Raw data (raw reads) from Illumina sequencing were processed using the Trimmomatic software. The reads containing the ploy-N and the low-quality reads were removed to obtain the clean reads. Then the clean reads were mapped to reference genome using hisat2. Fragments Per Kilobase of transcript per Million fragments mapped reads (FPKM) value of each gene was quantified using cufflinks software, and the read counts of each gene were obtained by htseq-count software. DEGs were identified using the DESeq (2012) R package with the threshold of *p*-value < 0.05 and fold Change (FC) > 2 or fold Change < 0.5. Volcano plots and hierarchical cluster analysis of DEGs was performed to explore the genes expression pattern. Gene ontology (GO) enrichment and KEGG pathway enrichment analysis of DEGs were performed using R package based on the hypergeometric distribution, respectively.

### Proteomics Analysis

*Saccharomyces cerevisiae* grown in YPD medium as mentioned above for 24 h. Pellets cells were lysed by using FASP buffer containing a complete protease inhibitors cocktail and phosphorylation protease inhibitors (Roche, Switzerland). The lysates were sonicated in same conditions and centrifuged to remove cellular debris. The supernatant was transferred to a fresh tube and the protein concentration was determined by 2-D Quant Kit (GE Healthcare, United States) (Picotti et al., 2009).

A total of 200 μg aliquots of protein were washed with 8 M urea, reduced with 20 mM DTT for 4 h at 37°C, and alkylated with 50 mM IAA for 30 min in the dark. Samples were resolubilized with 50 mM ammonium bicarbonate (NH_4_CO_3_) and digested with sequencing grade trypsin (Promega) to a final ratio of 1:50 (enzyme to substrate) for 16 h at 37°C. Peptide mixtures were lyophilized and labeled with 4-plex iTRAQ reagent (Sciex). Approximately 85 μg peptide samples of different oxygen concentrations (e.g., 21, 10, 5, and 1%) were labeled by iTRAQ 116, 118, 119, and 121, respectively. 70 μL of isopropanol was added into each sample and incubated in the dark for 2 h at room temperature. Then the labeled samples were combined into one tube and lyophilized. The peptide mixtures of different groups were cleaned up by Sep-Pak HLB cartridge (Waters, United States), eluted with 50% acetonitrile (ACN) containing 0.1% trifluoroacetic acid (TFA), and then dried by SpeedVac according to the previous study (Liu et al., 2017).

Samples were fractionated by high performance liquid chromatograph (HPLC) system (Agilent 1260 HPLC). The solvent consisted of 98% water and 2% ACN (pH 10) as mobile phase A and 98% ACN and 2% water (pH 10) as mobile phase B. The dried peptide mixture was reconstituted with 40 μL of mobile phase A and loaded onto a column (5 μm, 300 Å) at 42°C. The peptides were eluted at a flow rate of 0.7 ml/min. The separation gradient was set as follows: 0–30 min, 5–35% B; 30–32 min, 35–95% B; 32–37 min, 95% B; 37–39 min, 95–5% B; and 39–45 min, 5% B. Forty-five fractions were collected and pooled into nine fractions. Finally, fractions were dried by SpeedVac and stored at −80°C until LC-MS/MS analysis.

Chromatographic separations of the peptides were performed on an EASY-nLC system (Thermo Fisher, United States) with a C18 column (75 μm × 15 cm, 3 μm, 100 Å). The peptide mixture (0.5 μg) was separated in mobile phase A (0.1% formic acid in water) and mobile phase B (0.1% formic acid in ACN) at a flow rate of 300 nL/min. The separation gradient was set as follows: 8% B for 4 min, 30% B for 45 min, 90% B for 30 min, and 90% B for 3 min.

Mass spectrometry (MS) analysis was conducted on a Fusion Lumos Tribrid mass spectrometer (Orbitrap, Thermo Fisher, United States). The most abundant precursor ions and fragmentation data was acquired by HCD. The survey scans were acquired at a resolution of 15,000/30,000 at m/z 200. The precursor ions and product ions were scanned from 350 to 1550 Da and started from 120 m/z, respectively. The parameters of MS were set as follows: normalized collision energy, 31 eV; isolation width, 1.6 Da; capillary temperature, 300°C; dynamic exclusion duration, 20 s; and spray voltage, 2.0 kV.

The MS/MS spectra were searched using the MASCOT software (Matrix Science) with Proteome Discoverer 2.2. The following searching parameters were used to identify proteins: MS/MS tolerance, 0.1 Da; peptide mass tolerance, 20 ppm; enzyme, trypsin; enzymatic hydrolysis sites, arginine (R) and lysine (K); missed cleavage, 2; fixed modification, carbamidomethyl (C); variable modification, oxidation (M); and FDR, 1%.

The protein data was transformed by Perseus 1.6 software. Then global protein expression patterns and heatmap were illustrated using K-means clustering in Mev 4.9. The heatmaps of gradually up- or down-regulated in DEPs were also illustrated using HemI 1.0. The functional annotations and pathway enrichment analysis were performed by DAVID 6.8 and KEGG, respectively.

### Phosphoproteomics Analysis

Proteins were extracted and digested to peptide mixtures as previously described. The peptide mixtures of different groups were also cleaned up, desalinized and dried by SpeedVac. The phosphopeptide was purified and enriched by Titansphere Phos-TiO Kit (Shimadzu, Japan). The peptide mixtures were loaded into spin tips, equilibrated, adsorbed and rinsed with buffer A (0.4% TFA in ACN) and buffer B (25% solution B in buffer A). The enriched phosphopeptide was eluted with 5% ammonium hydroxide and 5% pyrrolidine solution. The phosphopeptide was dried by SpeedVac and stored at −80°C until LC-MS/MS analysis. The analytical methods of samples and bioinformation were processed as above-mentioned, except the variable modification of searching proteins parameters was phosphorylation (P).

### Metabolomics Analysis

About 15 mg of *S. cerevisiae* in four different oxygen concentrations was centrifuged at 1000 *g* and 4°C after incubation and washed by cooled water. The samples were added to 1.2 mL of the precooling extraction reagent (acetonitrile: methanol: water = 40:40:20), and freeze-thaw cycles were repeated three times in liquid nitrogen and ice. Then the samples were centrifuged at 10,000 *g* for 10 min at 4°C and the supernatant was evaporated to dry by high purity nitrogen gas at 4°C for LC-MS analysis. The metabolites were re-dissolved in water and each sample was pooled for quality control (QC) sample.

LC-MS analysis was used by ultra-performance liquid chromatography (UPLC, Waters) coupled with Synapt G2 mass spectrometer (Waters) which was equipped with data independent analysis (DIA) mode. The mobile phase flow rate was 0.4 mL/min and consisted of 0.1% formic acid and ammonium formate in water (A), and acetonitrile (B). The gradient profile was set as follows: 0.1–20% B for 5 min, 20–40% B for 5 min, 40–64% B for 4 min, 64–74% B for 3 min, 74–100% B for 1 min, 100% B for 1 min, and 0.1% B for 3 min.

Metabolites were separated using the Acquity UPLC HSS T3 column (2.1 mm × 150 mm, 1.8 μm). The parameters for mass spectrometry were as followed: capillary, 1.0 kV for positive mode and negative mode; sampling cone, 40 V; source offset, 80 V; Cone gas flow, 50 L/h; desolvation gas flow, 800 L/h; source temperature, 120°C. The continuum data was collected from 50 to 1200 Da and scan rate was 0.2 s (Kim et al., 2013).

The MS/MS data were analyzed using Progenesis QI software (Nonlinear Dynamics, Newcastle, United Kingdom). The peaks of each sample were aligned and picked according to retention time and m/z. The replicating peaks were removed by normalization and adduct deconvolution. The DMs were identified in the YMDB, KEGG and Lipid Maps databases as the threshold of VIP > 1, *p*-value < 0.05 and FC > 1.5 or FC < 0.6. Principle components analysis (PCA) and OPLS-DA were performed using SIMCA-P (Umetrics, Umea, Sweden). Pathway enrichment analysis of metabolites was achieved by the Metaboanalyst 4.0.

### RT-qPCR

The mRNA of *S. cerevisiae* was extracted using TRIzol (Thermo Fisher, United States) and chloroform reagent. After centrifugation, the mRNA was precipitated with isometric isopropanol in the aqueous phase, and purified with 75% ethanol in DEPC water. The concentration and purity of mRNA was determined using a spectrophotometer (NanoDrop 2000). The reverse transcription and the quantitative polymerase chain reaction (qPCR) were performed using the ReverTra RT Master Mix Kit (Toyobo, Japan) and SYBR Green Master Mix (Thermo Fisher, United States) in the QuantStudio 5 Real-Time PCR System (Thermo Fisher, United States), following the manufacturer’s protocol. The mRNA levels were calculated using the 2^–ΔΔCt^ method after normalization with TDH3 as a housekeeping gene. Primer sequences are listed in Supplementary Table S1.

### Growth Rate of *S. cerevisiae*

Phosphatidylcholine was chosen to verify its role in hypoxic stress. Phosphatidylcholine (2 and 10 μg/mL) was added into the YPD medium. *S. cerevisiae* were grown for 24 h in a three-gas cell culture incubator under Hpx1 condition. The absorbance values were determined at 600 nm from 4 to 24 h by the spectrophotometry with five replicated cultures. The maximum growth rate was calculated by non-linear regression.

### Statistical Analysis

Data were expressed as mean ± SD (standard deviation). Statistical analyses were assessed by SPSS 19.0 (IBM, United States). Differences were considered significant when *p*-values were lower than 0.05.

## Results

### The Change of *S. cerevisiae* Pathology in Hypoxic Stress

**FIGURE 1:**
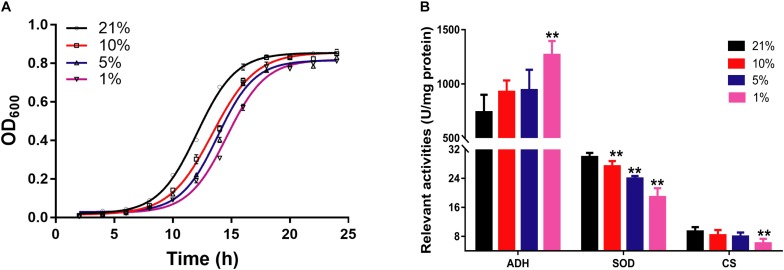
The pathophysiological changes of *S. cerevisiae* in hypoxic stress. **(A)** The OD_600_ values of cell at different times in different oxygen concentrations (*n* = 3, mean ± SD). **(B)** The enzyme activities of ADH, SOD, and CS (*n* = 7, mean ± SD). ^∗∗^*P* < 0.01: different oxygen concentrations compared with 21%.

The growth curve of *S. cerevisiae* in different oxygen concentrations was plotted according to the optical density at 600 nm (OD_600_) in Figure 1A. The growth curve of yeast cells had an obvious shift right with the decreasing of oxygen concentrations. In particular, the growth rate had marked difference from 10 to 18 h. The growth speed was significantly slower in hypoxic groups than that in the Con21 group.

The relevant enzymes activities were presented in Figure 1B. The activities of CS and SOD were decreased from 9.37 ± 1.21 to 6.11 ± 1.24 (*p* < 0.01) and from 29.90 ± 1.15 to 18.86 ± 2.46 (*p* < 0.01) between the Con21 and the Hpx1 group, respectively. However, the activities of ADH were obviously increased from 739.64 ± 161.04 to 1267.51 ± 128.94 between the Con21 and the Hpx1 group (*p* < 0.01).

### Transcriptomics Results

The numbers of DEGs consisted of up-regulation and down-regulation genes in different groups (Supplementary Data Sheet S1). Among the comparison of different oxygen concentrations, the DEGs were least in the Hpx5 compared with Hpx10, which includes 57 genes (40 up-regulated and 17 down-regulated genes). Besides, there were 128 DEGs (85 up-regulated and 43 down-regulated genes), 276 DEGs (210 up-regulated and 66 down-regulated genes) and 1017 DEGs (689 up-regulated and 328 down-regulated genes) with the decreasing of oxygen concentrations in the Hpx10, Hpx5, and Hpx1 compared with Con21, respectively.

**FIGURE 2:**
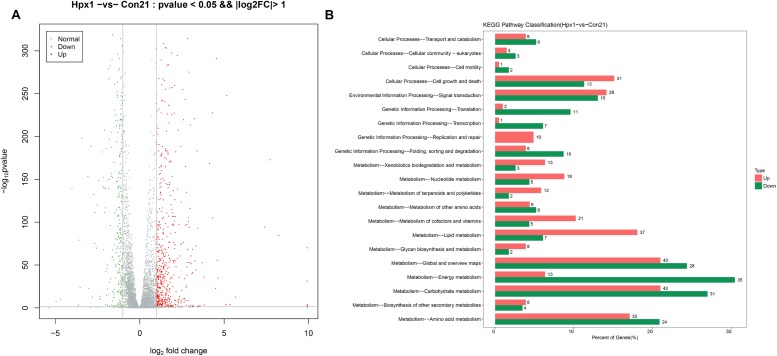
The mRNA expression levels and enrichment analysis in the transcriptomics. **(A)** The volcano plots of DEGs histogram of the number of DEGs between Hpx1 and Con21. **(B)** The distributing diagram of up-/down-regulated DEGs in KEGG pathway between Hpx1 and Con21. The red and green indicated up-regulated and down-regulated pathways, respectively. The numbers of beside column meant the numbers of DEGs in this pathway.

The numbers of DEGs between Hpx1 and Con21 was most different, and the disparity of oxygen concentration was also largest. Therefore, we displayed the data about these two groups as follows. Figure 2A showed the volcano plot between Hpx1 and Con21. The red was for significantly up-regulated DEGs (FC > 2) or high expressed genes, and green/blue was for notably down-regulated DEGs (FC > 2) or low expressed genes. The detailed information of DEGs and heatmaps between the Hpx1 and the Con 21 group were shown in Supplementary Data Sheet S1.

The results of GO enrichment analysis for DEGs were exhibited in Supplementary Data Sheet S1. The biological process of the up-regulated DEGs was mostly focused on FAs, ergosterol and DNA metabolic process, and the down-regulated DEGs were related to ATP synthesis coupled proton transport and tricarboxylic acid (TCA) cycle. The cellular component of the up-regulated DEGs were associated with various membranes and DNA polymerase, however, the down-regulated DEGs were chiefly related to nucleus and mitochondrion. The molecular function of the up-regulated DEGs were mostly focused to oxidoreductase activity, transferase activity and hydrolase activity, and the down-regulated DEGs was connected with transporter activity.

The pathways were sieved according to DEGs more than 2. The detailed pathways and top 20 bubble chart of up-/down-regulated DEGs between Hpx1 and Con21 were shown in Supplementary Data Sheet S1. The size and color of bubble indicated the numbers in pathway and *p*-value of DEGs, respectively. The up-regulated pathways were mostly involved in glycolysis/gluconeogenesis, steroid biosynthesis, pyruvate metabolism and tryptophan metabolism. The down-regulated pathways were largely focused on oxidative phosphorylation, biosynthesis of amino acids, pyruvate metabolism and TCA cycle.

Figure 2B showed the up-/down-regulated DEGs in KEGG pathway classification between Hpx1 and Con21. Among, the carbohydrate metabolic pathway significantly changed (43 up-regulated and 31 down-regulated genes), and the amino acid metabolic pathway visibly varied (35 up-regulated and 24 down-regulated genes). The energy metabolic pathway also extremely decreased with low hypoxic oxygen, containing 35 down-regulated and 13 up-regulated DEGs. However, the lipid metabolic pathway had a remarkable increase between Hpx1 and Con21, including 37 up-regulated and 7 down-regulated DEGs.

### Proteomics Results

**FIGURE 3:**
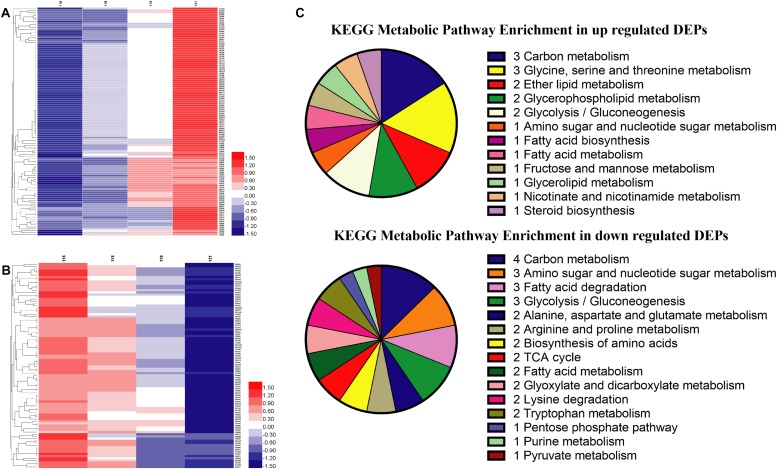
The proteins levels and enrichment analysis in the proteomics. **(A)** The heatmap of gradually up-regulated DEPs in different groups. **(B)** The heatmap of gradually down-regulated DEPs in different groups. The red indicated a high expression and the blue showed a low expression. **(C)** The distributing diagram of gradually up-/down-regulated DEPs in the metabolic pathway.

Global proteins were identified in four different oxygen concentrations to obtain 2412 proteins, and the heatmap was plotted in Supplementary Data Sheet S2. All proteins were screened with the threshold of *p*-value < 0.05 and FC > 1.2 or FC < 0.83 to gain 607 DEPs, 789 DEPs, and 214 DEPs in the results of Hpx1, Hpx5, and Hpx10 compared with Con21, respectively (Supplementary Data Sheet S2). The gradually up-/down-regulated DEPs were achieved with the decreasing of oxygen concentrations from Con21 to Hpx1 (Supplementary Data Sheet S2). Figures 3A,B were shown 119 gradually up-regulated proteins and 94 down-regulated proteins in four groups, respectively.

The results of GO enrichment analysis of 213 gradually up-/down-regulated DEPs were presented in Supplementary Data Sheet S2. In the data of gradually up-regulated DEPs, many biological processes, such as transposition, metabolic process, DNA biosynthetic process, phosphodiester bond hydrolysis, and lipid metabolic process, were varied by the molecular function changing of reaction (transferase, hydrolase, and catalytic enzyme) and binding reaction (RNA, metal ion, nucleotide, DNA, ATP, and nucleic acid) in the cytoplasm and nucleus. However, the biological processes in the gradually down-regulated DEPs were mostly related to translation, oxidation-reduction process, proton and electron transport, etc., and altered in the mitochondrion, membranes and ribosome. These DEPs activities of oxidoreductase, proton-transporting ATP synthase and cytochrome-c oxidase/reductase had obviously declined in the molecular function.

In the KEGG pathway analysis, metabolic pathways, ribosome, and oxidative phosphorylation were the main pathways in 213 gradually up-/down-regulated DEPs (Supplementary Data Sheet S2). Figure 3C showed the specific pathways in metabolic pathways, including carbohydrate, amino acids, FAs, and lipid metabolic pathways, etc. There were five marked proteins (up-regulated proteins: GPM2, sedoheptulose 1,7-bisphosphatase; down-regulated proteins: aldehyde dehydrogenase, ADH, pyruvate decarboxylase isozyme 1) in the glycolysis/gluconeogenesis pathway. However, proteins in partial pathways were only gradually down-regulated, but there were no up-regulated proteins in TCA cycle (e.g., CS, KGD2), purine metabolism (e.g., ribose-phosphate pyrophosphokinase), or pyruvate metabolism (e.g., aldehyde dehydrogenase). The serine metabolic pathway contained up-regulated proteins, but some proteins in the alanine, arginine, lysine, and tryptophan metabolic pathways were decreased in hypoxia stress. Some proteins in fatty acid biosynthesis metabolic pathway was up-regulated. The GPL metabolism and ether lipid metabolic pathways had two obviously improved proteins (e.g., lysophospholipid acyltransferase and EPT1).

### Phosphoproteomics Results

The parameters of phosphoproteomics were similar as described in proteomics. As found, 2826 phosphoproteins were identified in different groups (Con21, 2496; Hpx10, 2428; Hpx5, 2423; Hpx1, 2386) (Supplementary Data Sheet S3). 100 communal phosphoproteins were screened between 213 gradually up-/down-regulated proteins and Hpx1 phosphoproteins by Venny 2.1 (BioinfoGP, CNB-CSIC, Spain) (Supplementary Data Sheet S3).

**FIGURE 4:**
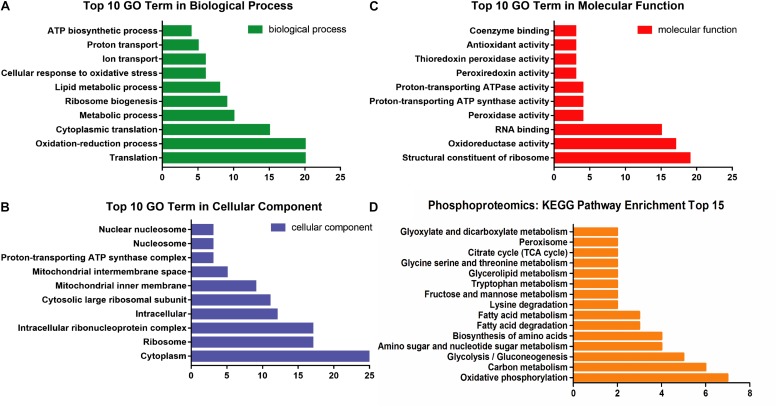
The enrichment analysis of 100 communal phosphoproteins. **(A)** The top 10 GO term in biological process. **(B)** The top 10 GO term in cellular component. **(C)** The top 10 GO term in molecular function. **(D)** The distributing diagram of KEGG pathway enrichment.

The GO analysis of 100 communal phosphoproteins was performed, including biological process, cellular component, and molecular function (Figures 4A–C). There were changes of biological process in the cytoplasm, ribosome, and mitochondrion, including transposition, oxidation-reduction, ribosome biogenesis, lipid metabolism, and transport process. The molecular function of significantly changed proteins were focused on the ribosome, oxidoreductase, RNA binding, peroxidase, ATP synthase and ATPase, etc.

These phosphoproteins were involved in 25 metabolic pathways and 15 ribosomes. Among the metabolic pathways, oxidative phosphorylation, carbohydrate (e.g., glycolysis and TCA cycle), amino acids (e.g., serine, lysine, tryptophan), FAs, and glycerolipid metabolic pathways were significantly changed and paralleled to the DEPs (see Figure 4D).

### Metabolomics Results

**FIGURE 5:**
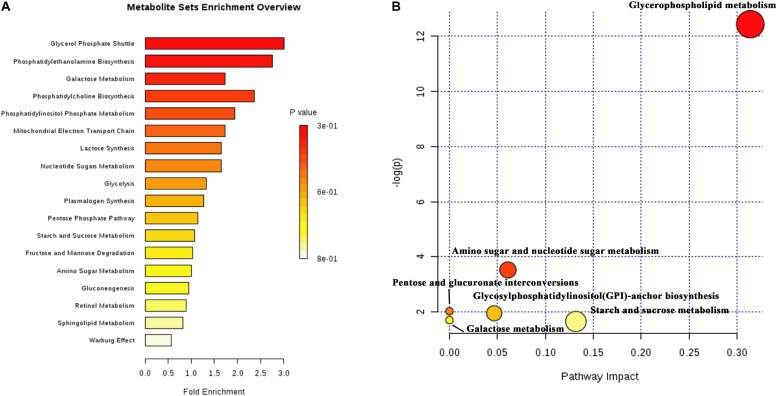
The results of differential metabolites and enrichment analysis in metabolomics. **(A)** The distributing diagram of metabolites enrichment. **(B)** The bubble chart of KEGG pathway enrichment. The red indicated a low *P*-value. The size of bubble indicated the numbers of DMs.

A total of 51 DMs were identified from the database and processed in the KEGG pathway enrichment analysis. The functions of these metabolites included the glycerol phosphate shuttle, PE biosynthesis, galactose metabolism, phosphatidylcholine (PC) biosynthesis, PI phosphate metabolism, and mitochondrial electron transport chain, etc (Figure 5A). The bubble chart of pathway enrichment contained the GPL metabolism, amino sugar and nucleotide sugar metabolism, glycosylphosphatidylinositol-anchor biosynthesis, and starch and sucrose metabolism, etc (Figure 5B). Among these pathways, the GPMP was significantly changed, including 34 DMs such as PC, PE, PI, PS, and their derivatives.

### Integration of Multi-Omics Data

**FIGURE 6:**
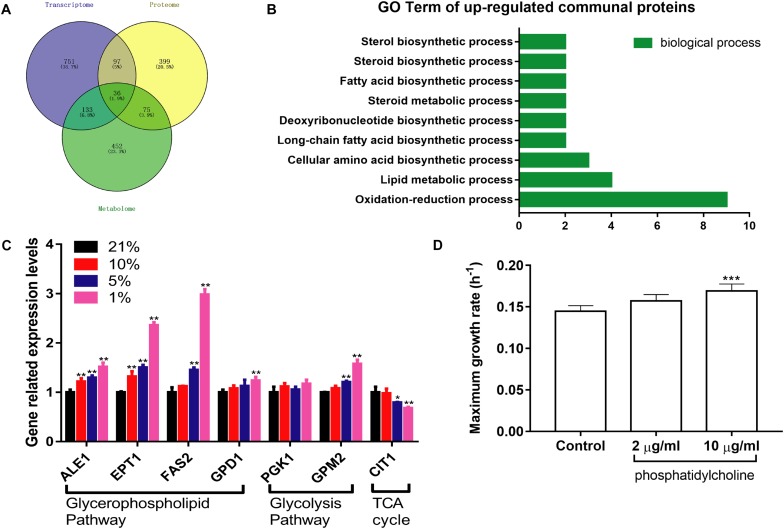
The integrated analysis and verification of multi-omics data. **(A)** The Venn diagrams of communal and key proteins in transcriptomics, proteomics, and metabolomics. **(B)** The biological process of GO analysis of 19 up-regulated communal and key proteins. **(C)** The expression levels of relevant mRNA in different oxygen concentrations (*n* = 3, mean ± SD). ^*^P < 0.05, ^∗∗^*P* < 0.01: different oxygen concentrations compared with 21%. **(D)** The maximum growth rate of PC at different concentrations under the Hpx1 condition (*n* = 5, mean ± SD). ^∗∗∗^*P* < 0.001: 10 μg/mL PC compared with the control group.

1017 DEGs were converted into downstream proteins between the Hpx1 and the Con21 group using DAVID 6.8, and the DMs were transformed into 696 upstream regulated proteins by the YMDB and Mbrole 2.0 (Spain) (Supplementary Data Sheet S4). The exported proteins from the DEGs and DMs were processed with 607 DEPs using Venny 2.1 to obtain the communal and key proteins in the transcriptomics, proteomics and metabolomics (Figure 6A). A total of 36 intersectional proteins, including 19 up-regulated and 17 down-regulated proteins, were simultaneously changed in these three omics data (Supplementary Table S2). Among these 36 proteins, we had a great interest in up-regulated proteins. In the biological process of GO analysis of 19 up-regulated proteins, lipid metabolic, fatty acid biosynthetic and steroid biosynthetic process had significant up-regulation, as enriched by DAVID 6.8 (Figure 6B).

### Verification of RT-qPCR

RT-qPCR was performed to verify the mRNA expression levels at different oxygen concentrations. The results were mainly consistent with the transcriptomics and proteomics data in Figure 6C. The mRNA levels of ALE1, ETP1, FAS2, and GPD1 were remarkably increased following the decline of oxygen concentrations from Con21 to Hpx1 in the GPMP. The GPM2 level was also enhanced, however, the PGK1 level was weakly changed in glycolysis pathway. Meanwhile, the CIT1 level in TCA cycle had obviously decreased in Hpx1 compared with Con21.

### Growth Rate of *S. cerevisiae*

After adding 2 and 10 μg/mL of phosphatidylcholine into the YPD medium under Hpx1 condition, the maximum growth rates were increased by 8.43% from 0.1448 h^–1^ ± 0.0068 to 0.1570 ± 0.0079 h^–1^ and 16.73% from 0.1448 h^–1^ ± 0.0068 to 0.1690 h^–1^ ± 0.0085 (*p* < 0.001), respectively (Figure 6D). Phosphatidylcholine (PC), one of the GPLs in DMs, promoted the growth of yeast cells under hypoxia and significantly increased the maximum growth rate.

## Discussion

Our study showed that the integrated approaches of multi-omics strategies were powerful for the analyses of the response of *S. cerevisiae* to hypoxic stress. The data from the transcriptome indicated that there were 1017 DEGs between Hpx1 and Con21 and most of them were listed in the glycolysis, TCA cycle, amino acid and lipid metabolic pathways. A total of 213 gradually up-/down-regulated DEPs, involved in carbohydrate, amino acids, FAs and lipid metabolic pathways, were discovered in proteome. 2386 proteins had phosphorylated modification under Hpx1 stress in phosphoproteome. Metabolome revealed that 51 DMs were identified between hypoxia and normoxia.

From a highly synergistic integration of multi-omics of hypoxic *S. cerevisiae* model, we found that the biological process of 19 up-regulated communal and key proteins mostly focused on lipid, fatty acid and steroid biosynthetic processes (Figure 6B). Moreover, the metabolites are the final products of the signaling pathways. The results of metabolomics were the closest to the phenotype of hypoxia, and reflect the real-time physiological state of organism. The majority of DMs were also related to lipid substances, and similar to the results of biological process of 19 up-regulated communal and key proteins. Among the DMs, the GPLs were the most obvious (Figure 5B). Hence, the GPMP was chosen to verify its role for growth of yeast cells in hypoxia. The results of RT-qPCR were confirmed and paralleled to the data of omics (Figure 6C). PC also significantly increased the maximum growth rate under hypoxia condition (Figure 6D). These results indicated that GPL increased the growth of yeast cells and reduced the cell damage induced by hypoxia.

**FIGURE 7:**
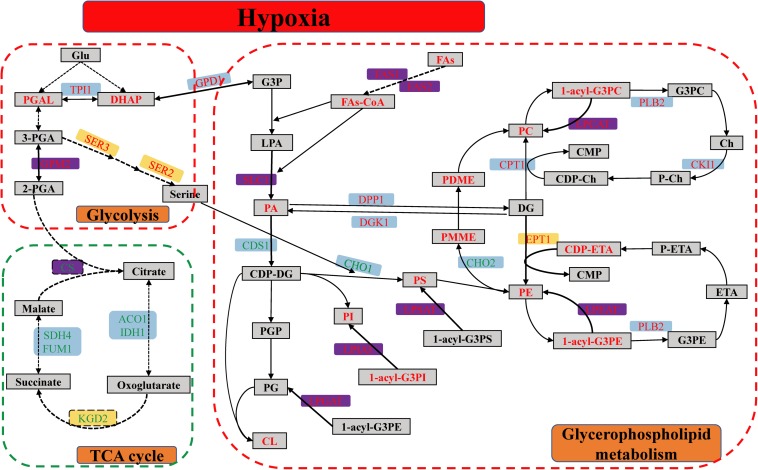
The metabolic pathway of glycerophospholipid, glycolysis, and TCA cycle of *S. cerevisiae* under hypoxia stress. The red dashed line box was up-regulated pathway, including GPL and glycolysis pathway, and the green dashed line box was down-regulated pathway of TCA cycle. The differentially expressed genes were indicated in the blue background. The differentially expressed proteins were showed in the yellow background. Both of DEGs and DEPs were indicated in the purple background. The metabolites were showed in the gray squares. The red and green word in different background indicated the up-regulated and down-regulated genes, proteins, or metabolites, respectively.

In detail, the GPMP were remarkably up-regulated between hypoxia and normoxia, including seven DEGs, two DEPs and thirty-four DMs. SLC1-encoded proteins catalyzed the reaction of 1-acyl-G3P (LPA) with FAs-CoA form 1,2-diacyl-G3P (PA). EPT1-encoded proteins catalyzed the reaction of CDP-ETA/CDP-Ch with DG form PE/PC. ALE1-encoded lysophospholipid acyltransferases contained LPCAT, LPEAT, LPSAT, LPIAT, and LPGAT enzyme was used for the synthesis of PC, PE, PS, PI, and PG, respectively (see Figure 7). The glycolysis pathway, including eighteen DEGs, two DEPs and metabolic intermediates, was also significantly up-regulated in different omics. This pathway provides energy and intermediates for cell reproduction and survival.

Glyceraldehyde 3-phosphate of DMs was transformed into DHAP subsequently to synthesize relevant GPL. Besides, the GPM2, SER3, and SER2-encoded protein were increased and played a key role in the synthetic process of serine. Serine was used for PS synthesis with CDP-DG (Figure 7). However, the biological process of oxidative phosphorylation was dramatically down-regulated, because the function of mitochondria was completely inhibited in the hypoxic environment. There were fifteen DEGs and DEPs in these conditions, respectively. Meanwhile, TCA cycle containing the nine DEGs and two DEPs had markedly down-regulated as well. CS was the rate-limiting enzyme of TCA cycle and decreased in mRNA and protein levels.

The metabolites of up-regulated GPMP included the phospholipids (PC, PE, PS, PI, PG, and CL), intermediates (LPA, PA, DG, CDP-DG, CDP-Ch, CDP-ETA, PMME, PDME) and minor molecule compounds such as ETA, Ch and FAs (Figure 7). These GPL composed the main lipid category of biological membranes and maintained the stability of cell membranes in hypoxia stress (Henry et al., 2012).

Glycerol 3-phosphate and FAs were the essential components of PA which play a key module in the regulation of various GPL (Ecker and Liebisch, 2014). G3P was originated from DHAP of glycolysis. The content of FAs was enhanced by FAS2-encoded fatty acid synthase in the mRNA and protein levels, and the acyl-CoA was synthesized with FAs form PA by ACB1-encoded protein (Figure 7). The degree of UFAs or the number of double bonds in the sn-1/sn-2 position of GPL was closely connected with the ability of antioxidant and protected unsaturated membrane lipids against oxidation induced by reactive oxygen species (ROS) (Broniec et al., 2017). In the hypoxic *S. cerevisiae*, the transcription factor MGA2 activated the transcription of OLE1 and itself to increase the UFAs and regulated the lipid homeostasis of membranes in response to hypoxia (Burr et al., 2017). *S. cerevisiae* without MGA2 could not grow due to that the low UFAs levels resulted in aberrant membrane transport (Burr et al., 2017; Romero et al., 2018). The function of MGA2 was analogous to mammalian sterol regulatory element-binding protein (SREBP) transcription factor 1, which regulates GPL synthesis (Burr et al., 2016).

The hypoxic phenomena were frequently arisen in different cancer cells and various pathological processes such as hypertrophic cardiomyopathy (Krishnan et al., 2009), Alzheimer’s disease (Frisardi et al., 2011), and perinatal asphyxia (Sánchez-Illana et al., 2017). For example, GPL biosynthesis pathway were up-regulated in human hepatocarcinoma, cervical adenocarcinoma and embryonic kidney cells, while lysophosphatidic acid acyltransferase β (LPAATβ) and HIF1α were jointly overexpressed to improve cell viability under hypoxia (Triantafyllou et al., 2018). LPA, a bioactive GPL, enhanced the invasiveness and adaptation of ovarian cancer cells by the metabolic rearrangement under hypoxia (Ray et al., 2017). Other researches explained that in the breast cancer cells under hypoxia stress, the lipid droplet and the PUFAs accumulation against oxidative toxicity were increased and extended their survival rate (Kamphorst et al., 2013). PUFAs synthesized phospholipids incorporated into membrane to protect astrocytes from oxidative damage (Zhang et al., 2015; Zgórzyńska et al., 2017). When the GPL in medium declined, multiple cancer cells decreased the survival rate in hypoxia and strongly impaired tumorigenesis (Bensaad et al., 2014; Jarc et al., 2018). In addition, the peroxisome proliferator-activated receptor γ (PPARγ) and HIF1α in human and mouse cardiac hypertrophy activated FAs uptake and GPL biosynthesis genes, and increased glucose-to-GPL conversion via G-3P pathway followed by changing myocardial metabolism and forming heart disease (Krishnan et al., 2009). GPL and GPL-derivatives constituted the backbone of neural membranes and provided the suitable environment of fluidity and ion permeability in Alzheimer’s disease. These GPL may play a key role in neuroprotection and suggest new preventive or therapeutic options to slow the neurodegenerative processes (Frisardi et al., 2011). In the hypoxic-ischemic encephalopathy secondary to perinatal asphyxia of the neonatal piglet study, the Ch and related metabolites of plasma was regarded as biomarker profiles for improving the early assessment of the severity of the hypoxic impairment in the neonate (Kuligowski et al., 2017).

## Conclusion

To summarize, the present study showed that the levels of the mRNA, proteins and metabolites had significantly altered in the hypoxic stress by the multi-omics techniques. In particular, the GPL and glycolysis metabolic pathways had remarkably up-regulated, however, the TCA cycle and oxidative phosphorylation processes had extremely down-regulated. Among these metabolic pathways, the various mediators of GPMP attenuated the hypoxic damage and maintained the stability of cell membranes. The features of GPL could provide new approaches and ideas for clinical research with hypoxic stress, especially in the neurodegenerative diseases, cardiovascular diseases and tumors. Our findings in the hypoxic omics and integrated networks provided an important insight to reveal the molecular mechanisms of hypoxic stress.

## Data Availability

The data had uploaded to the SRA database in NCBI, and its SRA accession number is PRJNA530059 (https://www.ncbi.nlm.nih.gov/sra/PRJNA530059).

## Author Contributions

MX, XZ, ZH, XL, and PX conceived and designed the experiments. ZX, JL, YM, YW, and LL performed the experiments. ZX, XZ, YM, LL, and MX analyzed the data. ZX, XZ, and MX wrote the manuscript. ZH designed the RNA-seq experiment.

## Conflict of Interest Statement

The authors declare that the research was conducted in the absence of any commercial or financial relationships that could be construed as a potential conflict of interest.

## Abbreviations

2-PGA: 2-phosphoglycerate
3-PGA: 3-phosphoglycerate
ADH: alcohol dehydrogenase
CDP: cytidine diphosphate
Ch: choline
CK: choline kinase
CL: cardiolipin
CoA: coenzyme A
Con: control
CS: citrate synthase
DEGs: differentially expressed genes
DEPs: differentially expressed proteins
DG: diacylglycerol
DHAP: dihydroxyacetone phosphate
DMs: differential metabolites
DTT: dithiothreitol
EPT1: choline/ethanolamine phosphotransferase
ETA: ethanolamine
FAs: fatty acids
FASP: filter-aided sample preparation
FDR: false discovery rate
G3P: glycerol 3-phosphate
G3PC: glycerol-3-phosphocholine
G3PE: glycerol-3-phosphoethanolamine
GC: gas chromatography
Glu: glucose
GPL: glycerophospholipid
GPM2: phosphoglycerate mutase 2
GPMP: glycerophospholipid metabolic pathway
HCD: higher-energy collisional dissociation
HIF: hypoxic-inducible factor
HPLC: high performance liquid chromatography
Hpx: hypoxia
IAA: iodoacetamide
iTRAQ: isobaric tags for relative and absolute quantification
KEGG: kyoto encyclopedia of genes and genomes
KGD2: alpha-ketoglutarate dehydrogenase
LPA: lysophosphatidic acid (1-acyl-G3P)
LPCAT, LPEAT, LPGAT, LPIAT, LPSAT: lysophospholipid acyltransferase
OPLS-DA: orthogonal partial least-squares discriminant analysis
PA: phosphatidic acid (1,2-diacyl-G3P)
PC: phosphatidylcholine/lecithin
P-Ch: phospho-choline
PDME: phosphatidyl-*N*-dimethylethanolamine
PE: phosphatidylethanolamine
P-ETA: phosphor-ethanolamine
PG: phosphatidylglycerol
PGAL: 3-phosphoglyceraldehyde
PGP: phosphatidylglycerolphosphate
PI: phosphatidylinositol
PMME: phosphatidyl-*N*-methylethanolamine
PS: phosphatidylserine
PUFAs: polyunsaturated fatty acids
*S. cerevisiae*: *Saccharomyces cerevisiae*
SER2: phosphoserine phosphatase
SER3: phosphoglycerate dehydrogenase
SILAC: stable isotope labeling with amino acids in cell culture
SLC1: lysophosphatidate acyltransferase
SOD: superoxide dismutase
UFAs: unsaturation fatty acids
YMDB: yeast metabolome database.
