# Supplementary material for: Multiple-Omics Techniques Reveal the Role of Glycerophospholipid Metabolic Pathway in the Response of Saccharomyces cerevisiae Against Hypoxic Stress
Source: Front Microbiol. 2019 Jun 27;10:1398. doi: 10.3389/fmicb.2019.01398 (PMC6610297; doi:10.3389/fmicb.2019.01398)
Supplement: Supplementary file 1 [file Table_1.DOC]

***Supplementary Material***

Supplementary Table S1. Primers for real-time quantitative reverse transcription polymerase chain reaction.

| Gene name | Primer Sequences | Melting Temp (°C) |
| --- | --- | --- |
| ALE1 | F:5-GCGCAGAATACGCGTGAAAT-3 | 55.4 |
|  | R:5-TACAAAGCCCCTGTCGCAAA-3 | 55.4 |
| EPT1 | F:5-TCACGGGAGCATTTACCGTC-3 | 57.5 |
|  | R:5-AACCCAAGCCAAGATAGGGC-3 | 57.5 |
| GPD1 | F:5-CTGGCATCACTCTACCCGAC-3 | 59.4 |
|  | R:5-GCACCAGATAGAGCACCACA-3 | 57.4 |
| PGK1 | F:5-TGCCAAGGTTGCTGACAAGA-3 | 55.3 |
|  | R:5-GAACGATTTCAGCACCAGCC-3 | 57.4 |
| GPM2 | F:5-TGGAGACGAATTTGGCAGTGA-3 | 55.6 |
|  | R:5-TACAGGTGGTGGCTTACCCT-3 | 57.4 |
| TDH3 | F:5-ACTGTCCACTCTTTGACTGCT-3 | 55.6 |
|  | R:5-CGACGGTTGGGACTCTGAAA-3 | 57.4 |
| CIT1 | F:5-TTTGGCCGCTGGTTTGAATG-3 | 55.3 |
|  | R:5-GGAACAACTCTCCCTGCGTT-3 | 57.4 |
| FAS2 | F:5-CTGGTCATCCAAATGGGTGCT-3 | 57.5 |
|  | R:5-AAGTGATGGACACGGCTCTG-3 | 57.5 |
