# Supplementary material for: Multiple-Omics Techniques Reveal the Role of Glycerophospholipid Metabolic Pathway in the Response of Saccharomyces cerevisiae Against Hypoxic Stress
Source: Front Microbiol. 2019 Jun 27;10:1398. doi: 10.3389/fmicb.2019.01398 (PMC6610297; doi:10.3389/fmicb.2019.01398)
Supplement: Supplementary file 2 [file Table_2.DOC]

***Supplementary Material***

Supplementary Table S2. The communal proteins in transcriptome, proteome and metabolome.

| Accession ID | Expression levels (Hpx1/Con21) |
| --- | --- |
| P32466 | 2.174 |
| P22217 | 1.686 |
| P00635 | 1.578 |
| P32377 | 1.403 |
| Q04894 | 1.355 |
| Q06143 | 1.353 |
| P38858 | 1.351 |
| P40353 | 1.341 |
| P32614 | 1.338 |
| P53199 | 1.293 |
| P07149 | 1.285 |
| P19097 | 1.284 |
| P38145 | 1.263 |
| P18544 | 1.244 |
| P15454 | 1.238 |
| Q06489 | 1.234 |
| P09938 | 1.233 |
| P37012 | 1.231 |
| P40054 | 1.214 |
| P06169 | 0.831 |
| P04819 | 0.828 |
| P06208 | 0.822 |
| P32288 | 0.814 |
| P28241 | 0.785 |
| P52910 | 0.781 |
| P31688 | 0.775 |
| P21801 | 0.772 |
| P46367 | 0.722 |
| P27796 | 0.721 |
| P30952 | 0.721 |
| P21826 | 0.721 |
| Q07505 | 0.710 |
| P41940 | 0.697 |
| Q12166 | 0.686 |
| P39708 | 0.584 |
| P40215 | 0.457 |
