# Supplementary material for: Multiple-Omics Techniques Reveal the Role of Glycerophospholipid Metabolic Pathway in the Response of Saccharomyces cerevisiae Against Hypoxic Stress
Source: Front Microbiol. 2019 Jun 27;10:1398. doi: 10.3389/fmicb.2019.01398 (PMC6610297; doi:10.3389/fmicb.2019.01398)
Supplement: Supplementary file 3 [file Data_Sheet_1.zip › Data Sheet 1_Figure legends.docx]

**Figure legends**

**Figure 1.** The numbers of DEGs in four different groups.

**Figure 3.** The heatmap of DEGs between Hpx1 and Con21.

**Figure 6.** The top 30 GO terms of up-regulated DEGs between Hpx1 and Con21.

**Figure 7.** The top 30 GO terms of down-regulated DEGs between Hpx1 and Con21.

**Figure 12.** The top 20 KEGG pathways of up-regulated DEGs between Hpx1 and Con21.

**Figure 13.** The top 20 KEGG pathways of down-regulated DEGs between Hpx1 and Con21.
