# Supplementary figures and images for: Multiple-Omics Techniques Reveal the Role of Glycerophospholipid Metabolic Pathway in the Response of Saccharomyces cerevisiae Against Hypoxic Stress
Source: Front Microbiol. 2019 Jun 27;10:1398. doi: 10.3389/fmicb.2019.01398 (PMC6610297; doi:10.3389/fmicb.2019.01398)

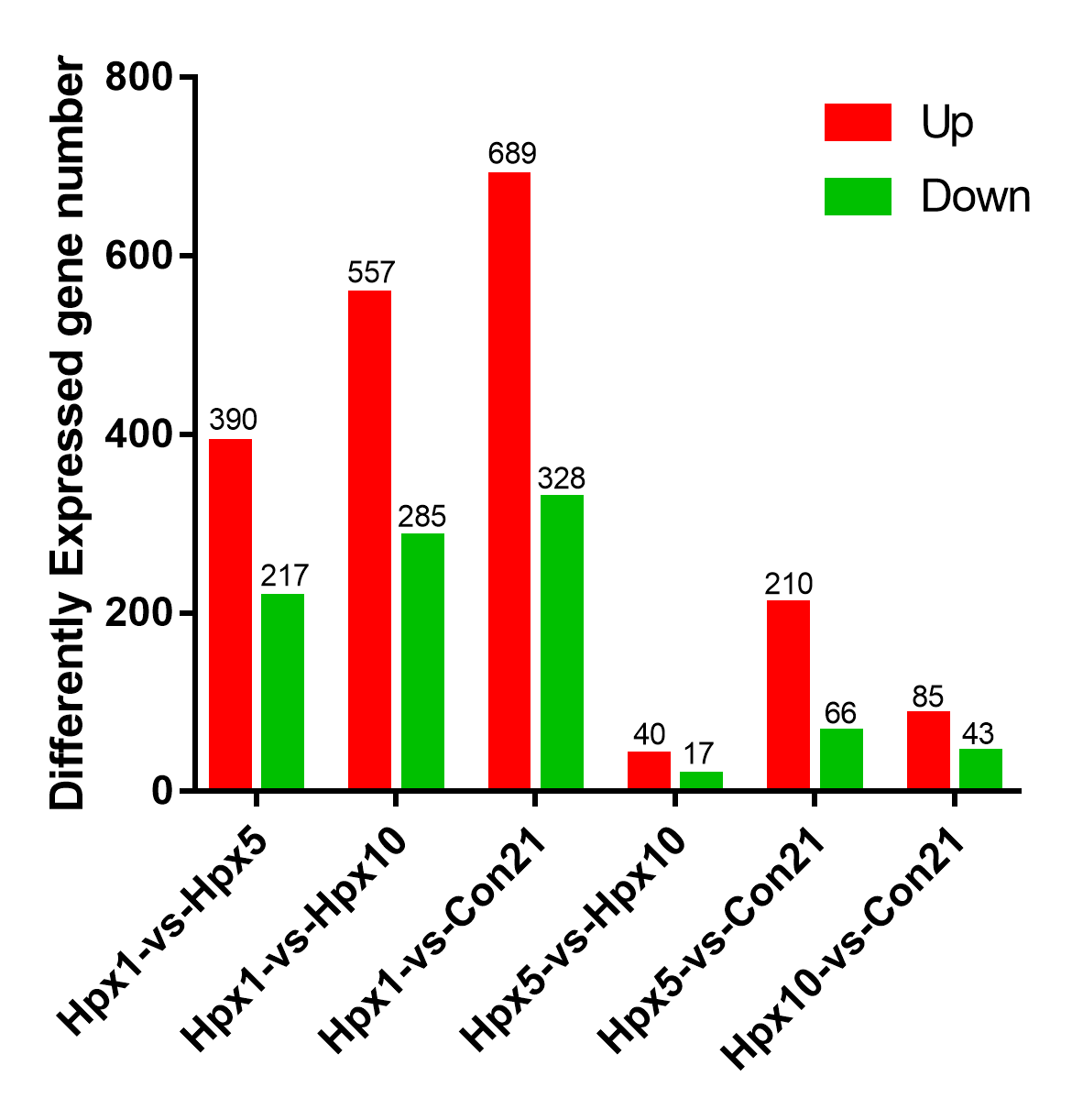

Supplement: Supplementary file 3 [file Data_Sheet_1.zip › 1.The numbers of DEGs.tif]

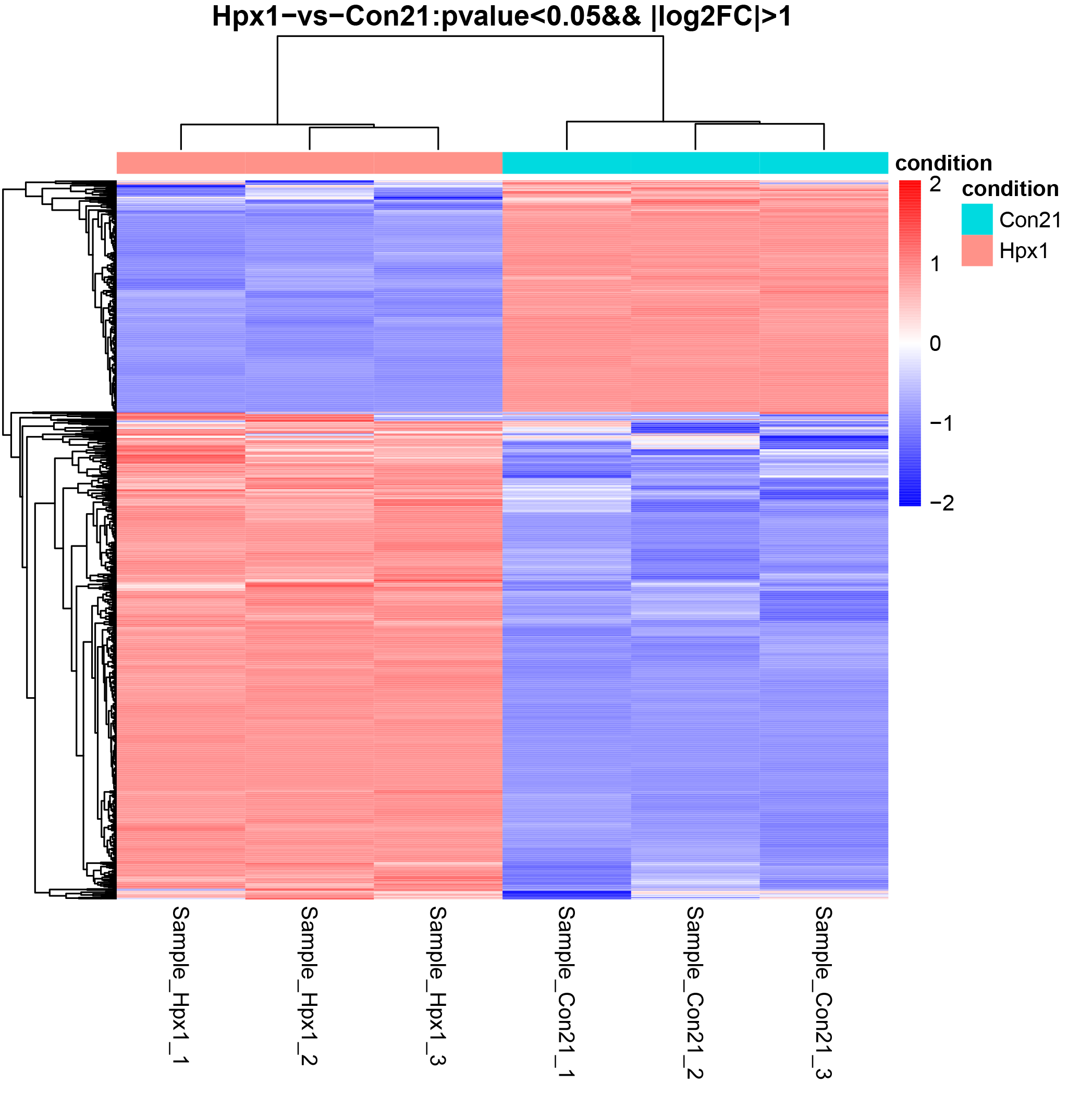

Supplement: Supplementary file 3 [file Data_Sheet_1.zip › 3. heatmap-Hpx1-vs-Con21.tif]

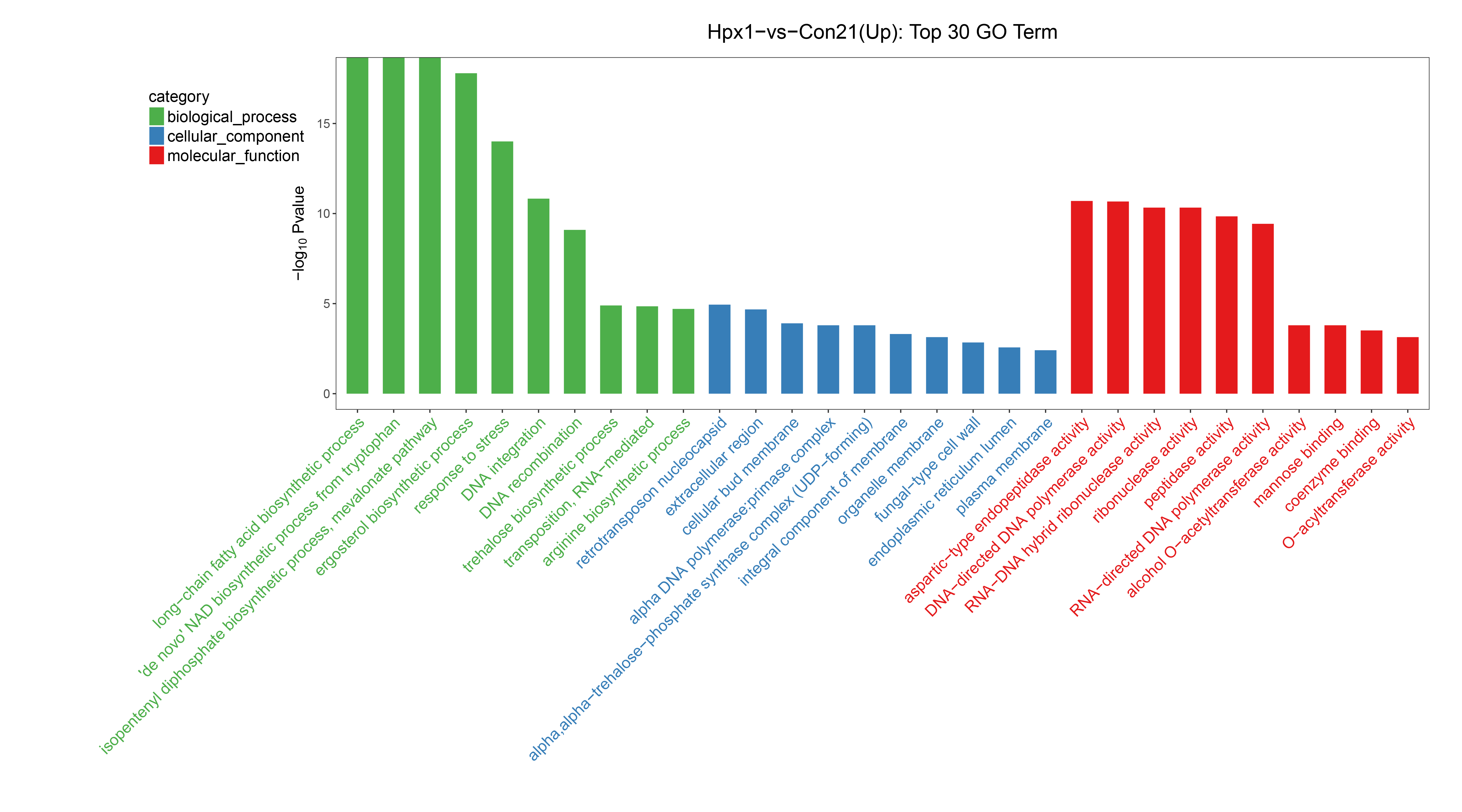

Supplement: Supplementary file 3 [file Data_Sheet_1.zip › 6. GO top30 up.tif]

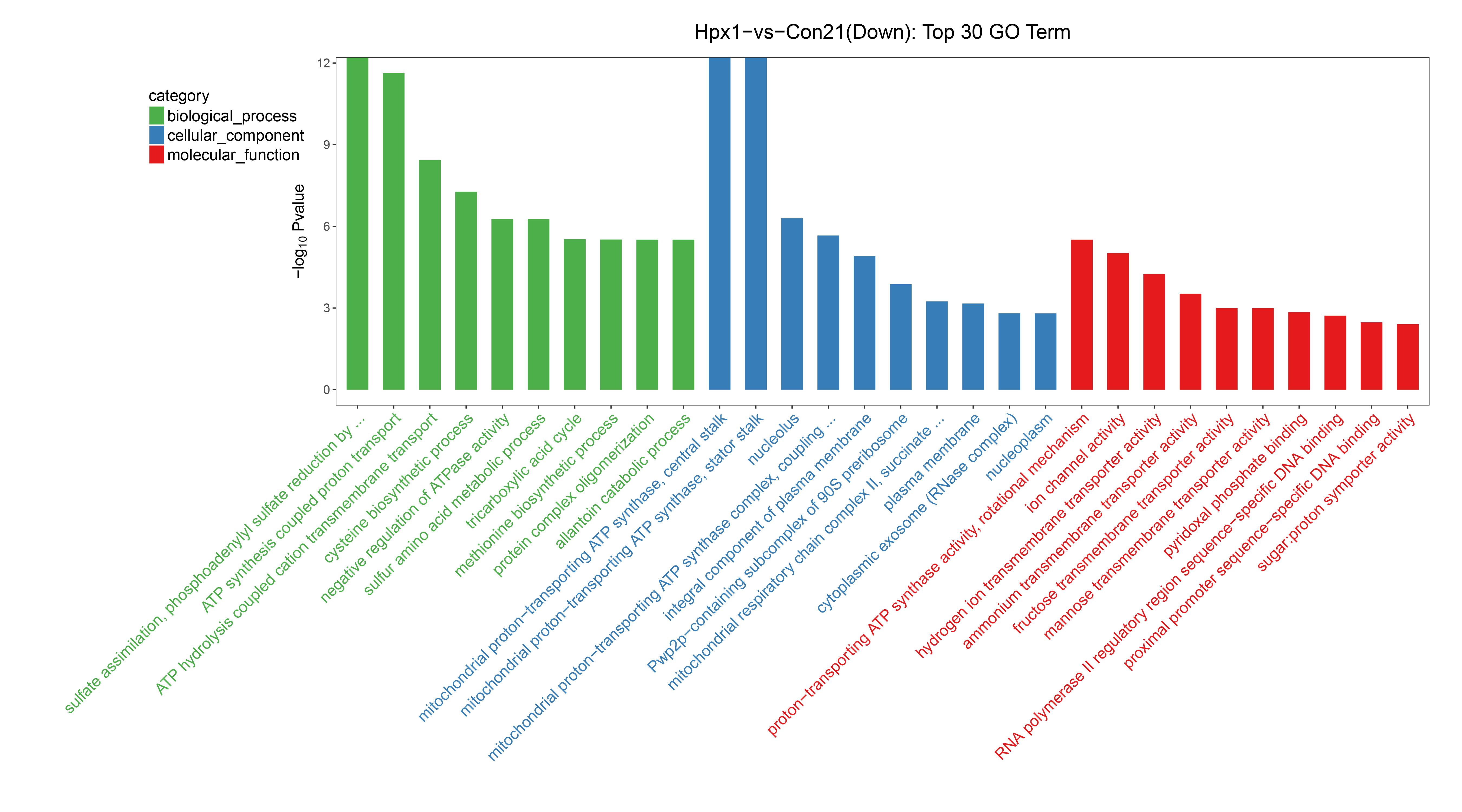

Supplement: Supplementary file 3 [file Data_Sheet_1.zip › 7. GO top30 down.tif]

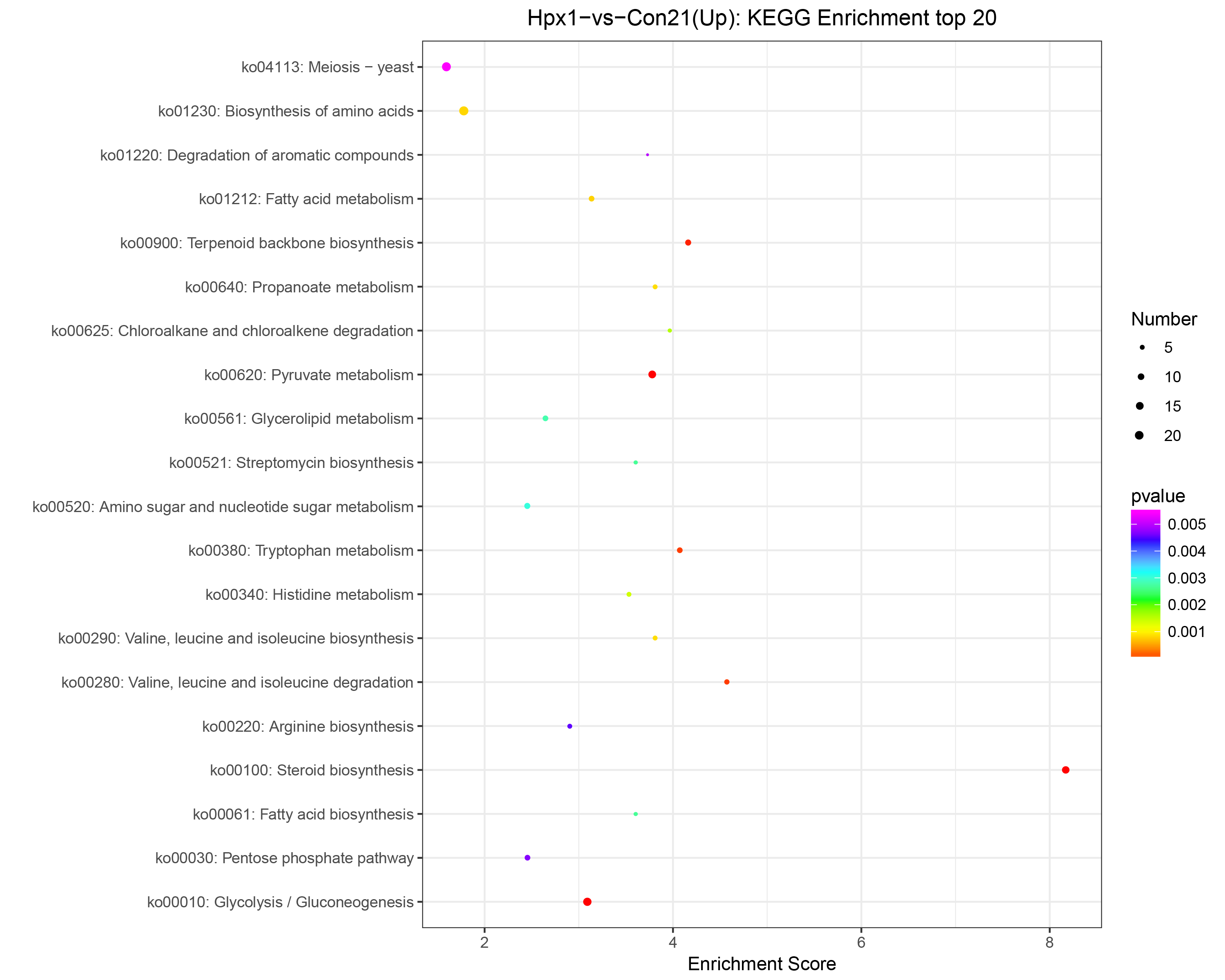

Supplement: Supplementary file 3 [file Data_Sheet_1.zip › 12. KEGG top20 up.tif]

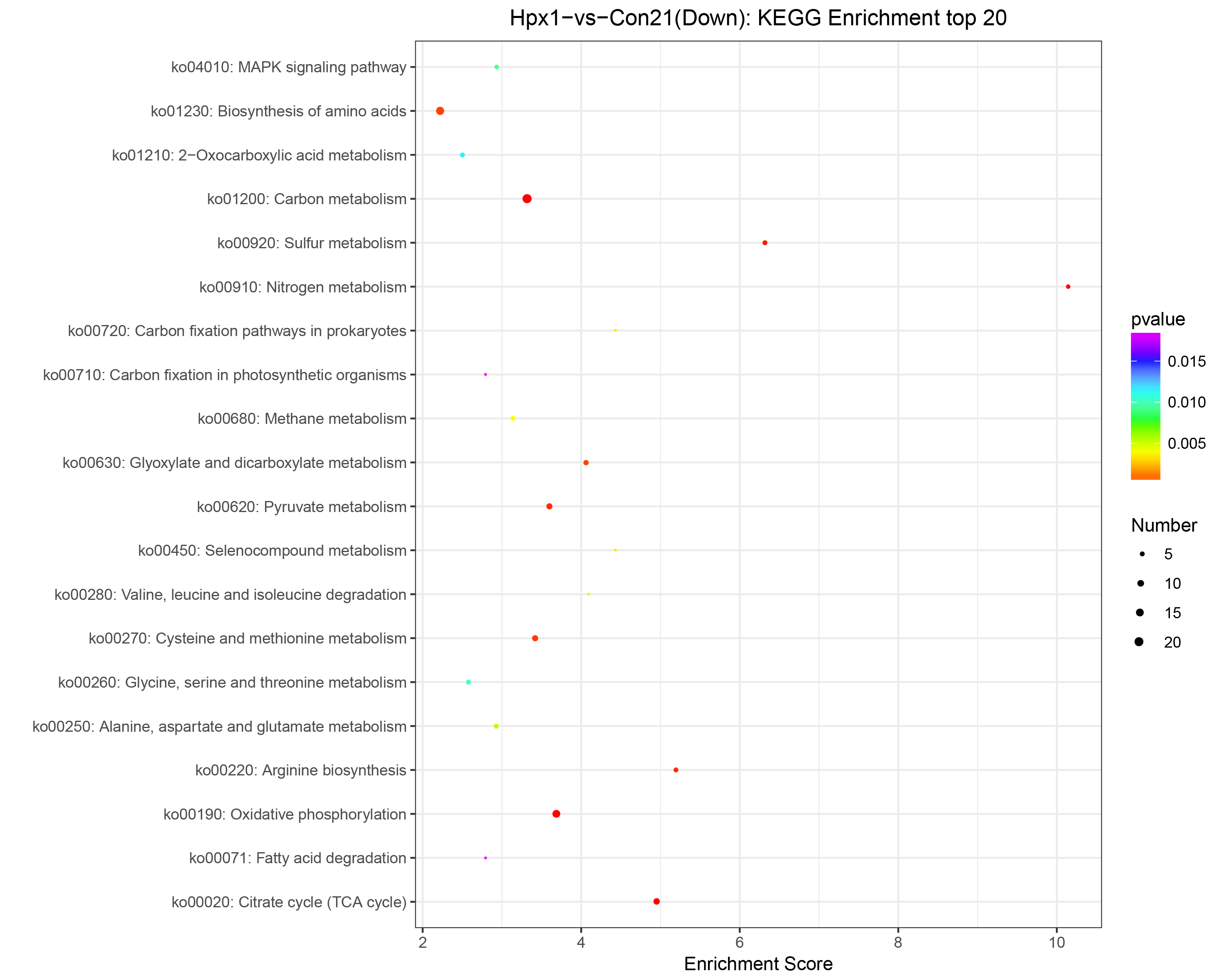

Supplement: Supplementary file 3 [file Data_Sheet_1.zip › 13. KEGG top20 down.tif]

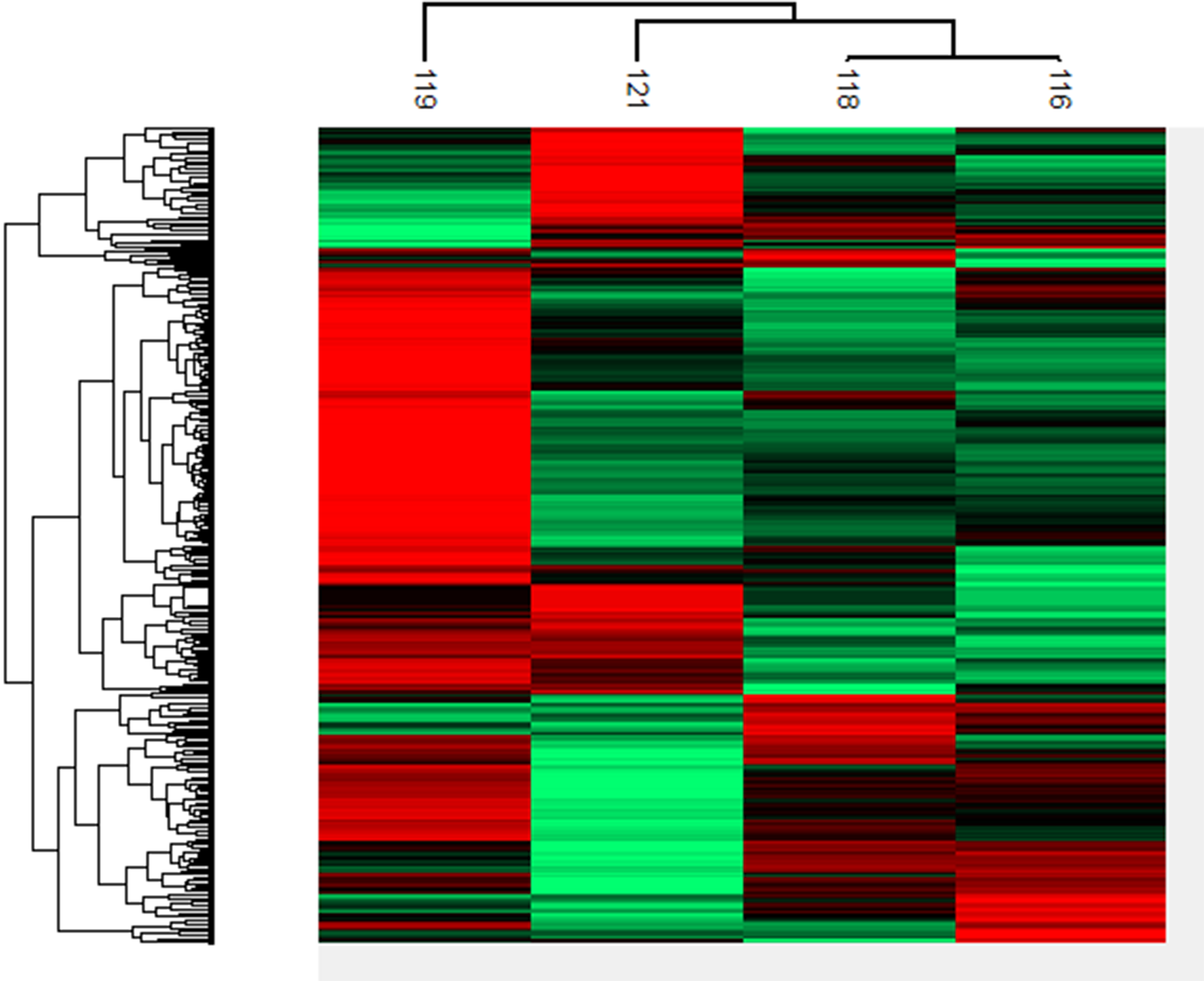

Supplement: Supplementary file 4 [file Data_Sheet_2.zip › 2. The heatmap of global proteins.tif]

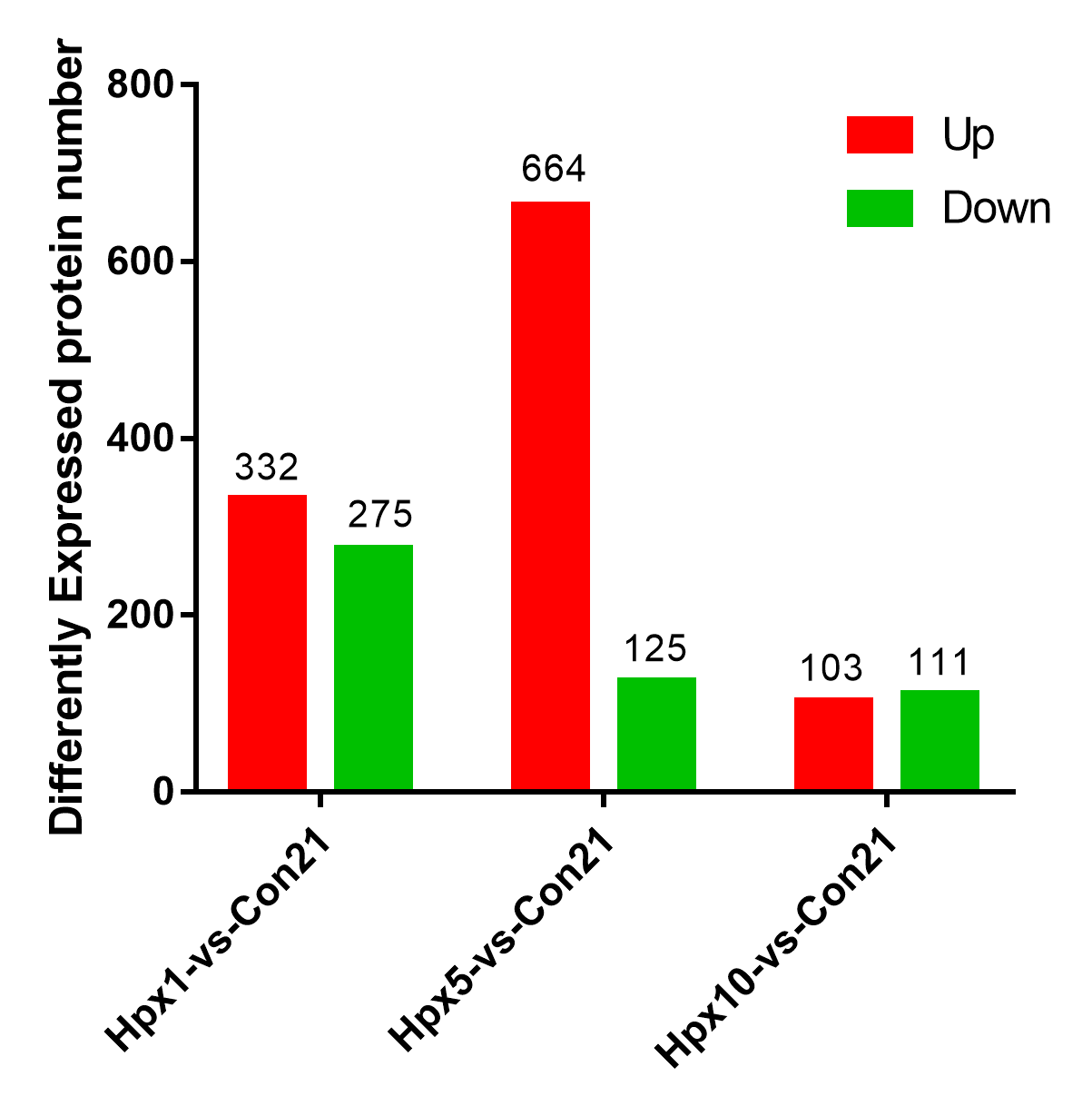

Supplement: Supplementary file 4 [file Data_Sheet_2.zip › 3. The numbers of DEPs.tif]

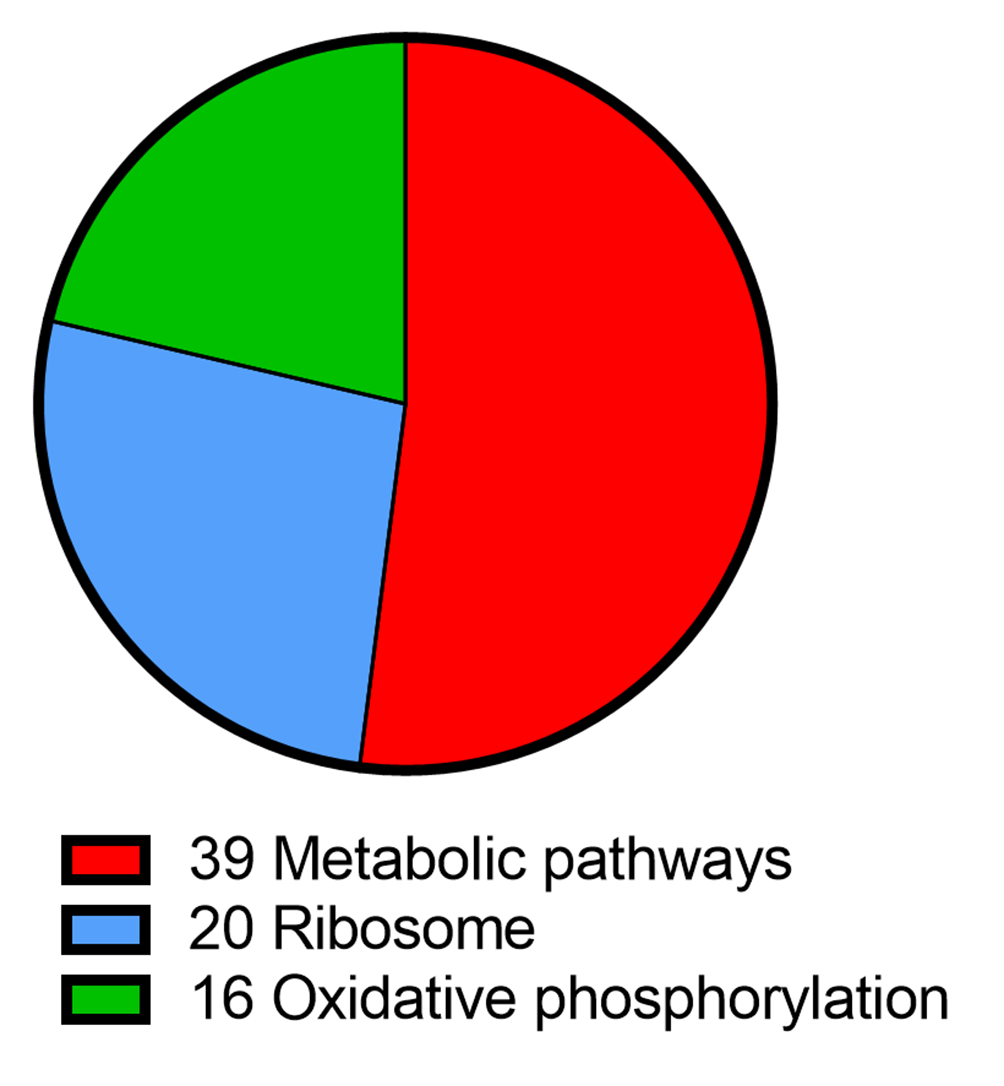

Supplement: Supplementary file 4 [file Data_Sheet_2.zip › 10. kegg pathways.tif]

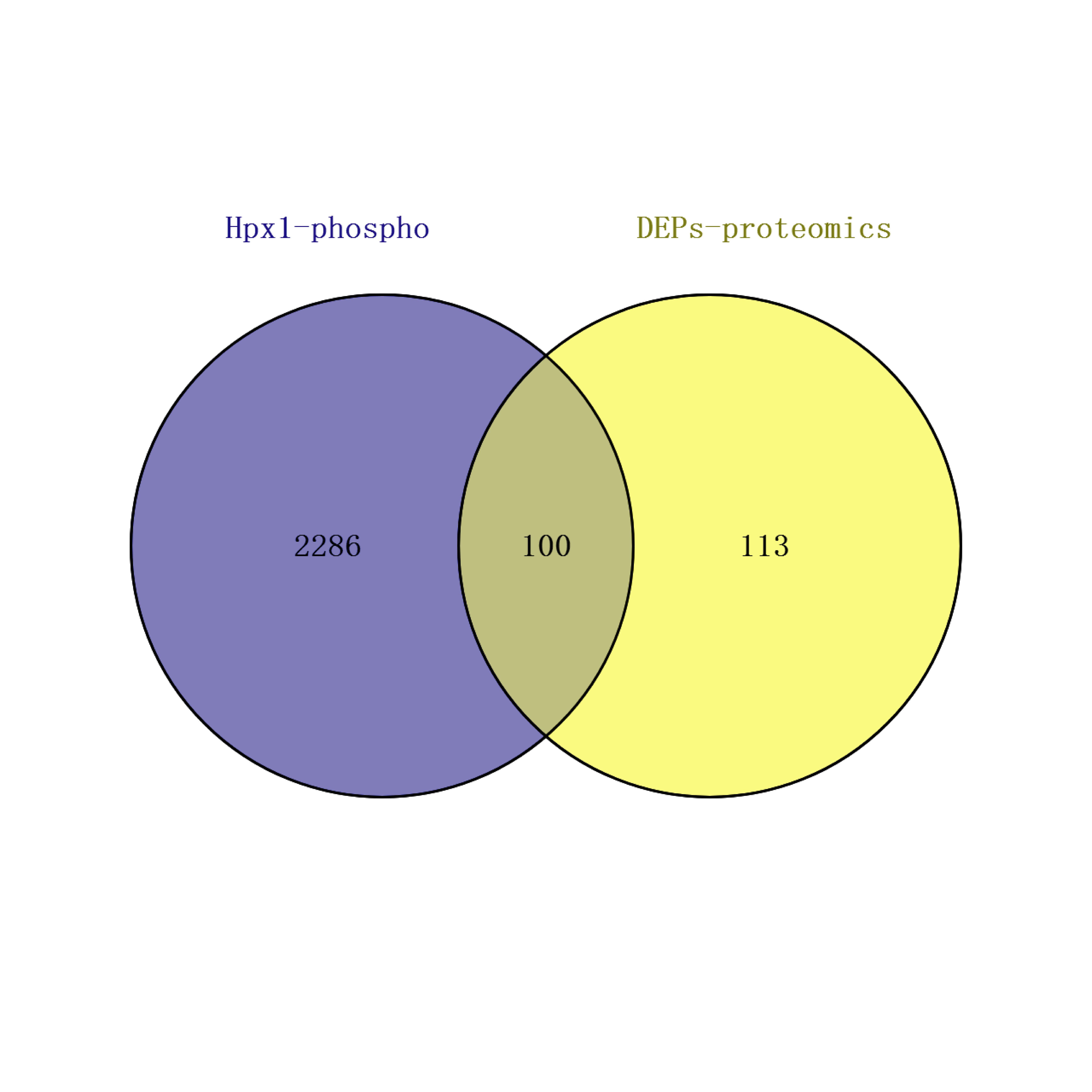

Supplement: Supplementary file 5 [file Data_Sheet_3.zip › 3. The venn diagram of 100 communal proteins.tif]
