# Supplementary material for: Multiple-Omics Techniques Reveal the Role of Glycerophospholipid Metabolic Pathway in the Response of Saccharomyces cerevisiae Against Hypoxic Stress
Source: Front Microbiol. 2019 Jun 27;10:1398. doi: 10.3389/fmicb.2019.01398 (PMC6610297; doi:10.3389/fmicb.2019.01398)
Supplement: Supplementary file 4 [file Data_Sheet_2.zip › Data Sheet 2_Figure legends.docx]

**Figure legends**

**Figure 2.** The heatmap of global proteins in four different groups.

**Figure 3.** The numbers of DEPs between Hpx and Con21.

**Figure 8.** The top 30 GO terms of gradually up-regulated DEPs between Hpx1 and Con21.

**Figure 9.** The top 30 GO terms of gradually down-regulated DEPs between Hpx1 and Con21.

**Figure 10.** The KEGG pathways of gradually up-/down-regulated DEPs between Hpx1 and Con21.
