# Supplementary material for: Multiple-Omics Techniques Reveal the Role of Glycerophospholipid Metabolic Pathway in the Response of Saccharomyces cerevisiae Against Hypoxic Stress
Source: Front Microbiol. 2019 Jun 27;10:1398. doi: 10.3389/fmicb.2019.01398 (PMC6610297; doi:10.3389/fmicb.2019.01398)
Supplement: Supplementary file 5 [file Data_Sheet_3.zip › Data Sheet 3_Figure legends.docx]

**Figure legends**

**Figure 3.** The Venn diagram between phosphoproteomics in Hpx1 and gradually up-/down-regulated DEPs.
