# Supplementary material for: Multiple-Omics Techniques Reveal the Role of Glycerophospholipid Metabolic Pathway in the Response of Saccharomyces cerevisiae Against Hypoxic Stress
Source: Front Microbiol. 2019 Jun 27;10:1398. doi: 10.3389/fmicb.2019.01398 (PMC6610297; doi:10.3389/fmicb.2019.01398)
Supplement: Supplementary file 6 [file Data_Sheet_4.zip › Data Sheet 4_Captions.doc]

**Captions**

**Table 1.** The Gene ID and Accession ID of the 1017 differentially expressed genes (DEGs).

**Table 2.** The Accession ID of the 696 differential metabolites (DMs).

**Table 3.** The Accession ID of the 607 differentially expressed proteins (DEPs).
